# Evidence of quantum-entangled higher states of consciousness

**DOI:** 10.1016/j.csbj.2025.03.001

**Published:** 2025-03-10

**Authors:** Álex Escolà-Gascón

**Affiliations:** Department of Quantitative Methods and Statistics, Comillas Pontifical University, erected by the Holy See, Vatican City State

**Keywords:** Consciousness, Nonlocality, Quantum-Multilinear Integrated Coefficient, Neuroplasticity, Anomalous, Psi phenomena

## Abstract

What if quantum entanglement could accelerate learning by unlocking *higher* states of conscious experience? This study provides empirical and statistical evidence of how quantum entanglement influences consciousness at a biophysical level. We analyzed data from 106 monozygotic twin pairs (*N* = 212), randomly assigned to control and experimental groups. Using a consanguinity-based matching technique, twin pairs (A-B) were formed. Two distinct 2-qubit circuits were designed: C1 (non-entangled) for the control group and E1 (entangled) for the experimental group. These circuits manipulated visual stimulus contingencies during a 144-trial implicit learning experiment conducted under nonlocal conditions, executed via the *IBM Brisbane* supercomputer. Mental states were assessed with 3D *electroencephalography* (EEG), while biomarkers—including *Brain-Derived Neurotrophic Factor* (BDNF) for neuroplasticity, *Free Fatty Acids* (FFA), and Alpha-Amylase for physiological arousal—were measured. To advance this field, we introduced the *Quantum-Multilinear Integrated Coefficient* (*Q*), a groundbreaking metric capable of estimating variance increases attributable to quantum entanglement effects within response matrices. Our findings revealed that the entanglement of qubits in stimulus configurations explained 13.5 % of the variance in accuracy within the experimental group. The *Q* coefficient captured up to a 31.6 % increase in variance across twin responses, while neuroplasticity markers explained a 26.2 % increase in cognitive performance under entangled conditions. These results provide robust evidence that quantum entanglement enhances conscious experience and facilitates faster, more efficient learning. They point to the existence of anomalous cognitive mechanisms capable of anticipating future, unpredictable stimuli, representing a profound leap in our understanding of consciousness and its quantum underpinnings.

## Highlights

• We tested whether qubit entanglement impacts implicit learning, neuroplasticity, and anomalous cognition.
• The new coefficient *Q* effectively estimates combined variance from quantum and non-quantum effects.
• Qubit entanglement synchronization optimizes stimulus contingencies, boosting learning efficiency.
• Our evidence supports the possibility of connecting human brains to quantum entanglement processes.
• We surpassed Bell's 2√2, revealing superquantum effects relevant to consciousness research.

## Graphical abstract

**Figure:**
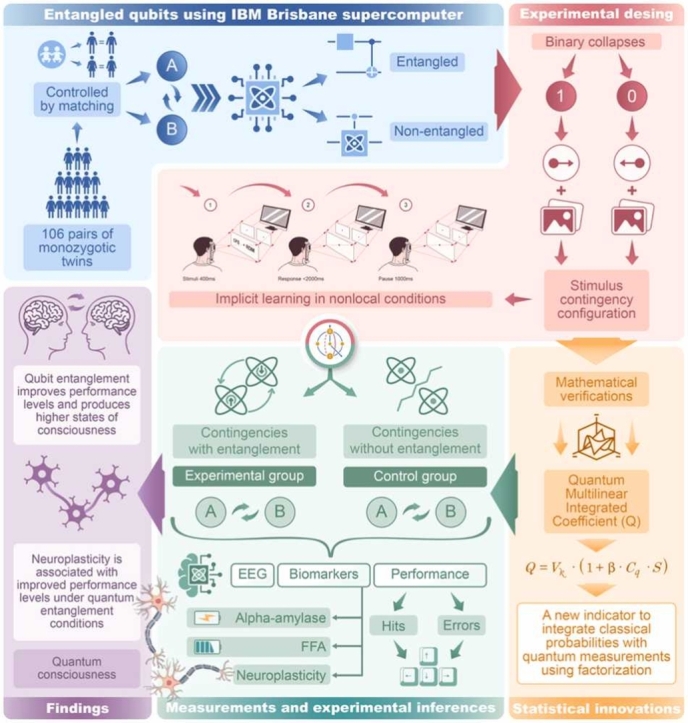
*Designed © by Prof. Dr. Álex Escolà-Gascón. All rights reserved.***Notice:** This figure, along with some of its illustrations or parts, was previously used in the Open Access publication by Escolà-Gascón (2024). The author of this report retains full reproduction rights and permission to reuse them in this article.

## 1 Introduction

The question of why a handful of neurons enable us to experience the sweetness of honey, the softness of a caress, or the warmth of a hug remains unresolved, despite current scientific advancements [55]. Furthermore, *Nature* has acknowledged that neuroscientists have yet to identify the source and origin of conscious sensory experiences or explain why consciousness manifests so differently in individuals exposed to identical sensory conditions [46]. Contemporary scientific debate focuses less on understanding *how* conscious perceptions provoke the mechanisms behind causal or concurrent factors to arise and more on the unresolved mystery of *why* we feel and why sensory experiences are so diverse [22]. Chalmers [15] famously termed this the *hard problem of consciousness,* which remains a central challenge in neuroscience and biology [8].

Given the persistent gaps in understanding the origins and mechanisms of conscious sensory experiences, it is reasonable to question the ontological boundaries of consciousness itself [81]. Acknowledging consciousness as a mystery compels scientists to rigorously explore hypotheses within orthodox limits while remaining open to ideas that push the frontiers of scientific inquiry [10]. One particularly challenging form of conscious experience at these frontiers is anomalous cognition [52].

### 1.1 Life requires cognition at all levels

Shapiro [74] famously asserted, *“life requires cognition at all levels,”* including the “anomalous.” Anomalous cognition refers to conscious experiences where organisms access remote, delocalized information within the space-time continuum through mechanisms currently unknown to science [72], [24]. This delocalization implies that such information is independent of time and space, accessible from locations unconnected to the receiver and spanning past, present, and future [79]. The term “anomalous” highlights the limited understanding of its origins and mechanisms, though ongoing research proposes that quantum principles may underpin these phenomena, offering promising explanations for these seemingly impossible experiences [21].

Among anomalous cognitions, precognition—the ability to anticipate future stimuli—is the most extensively studied. It has been hypothesized as being a biological mechanism for survival and environmental adaptation, functioning as a homeostatic resource to mitigate unpredictable dangers [56]. Within this context, precognition can be viewed as a conscious experience rooted in the evolutionary and synthetic framework of species [58], aligning with the *Cellular Basis of Consciousness* (CBC) model by Baluška and Reber [6]. The CBC posits that consciousness and emotions are molecular products of evolution rather than solely neural network activity [69]. It further suggests that all forms of life, from unicellular to multicellular organisms, possess basic consciousness and affective experiences [68].

This line of research is less speculative than it may seem. Segundo-Ortin and Calvo [73] demonstrated that *Phaseolus vulgaris* plants exhibited cognitive behaviors by accessing environmental information anomalously to guide growth decisions—actions that were neither reflexive nor random [67]. Similar findings highlight mimetic anomalous behaviors in other plant species [60]. Shapiro [75], providing evidence of primitive cognitive states in prokaryotic cells and presenting challenges to current epistemic frameworks that struggle to incorporate such findings [5].

Additionally, the orientation sense in migratory birds, enabling them to traverse vast distances and consistently follow identical routes, represents another anomalous biological phenomenon linked to consciousness and precognition [40]. Emerging research suggests quantum processes may underlie these behaviors, establishing a significant intersection between consciousness, biology, and quantum theory [41]. Collectively, this evidence and these theoretical frameworks suggest that anomalous cognition is multispecies in nature. As Ellia et al. [22] argue, it is the scientific community’s responsibility to investigate and elucidate the source, origins, development, and functionality of these seemingly impossible phenomena.

### 1.2 Quantum consciousness and cognition

Neuroscience recognizes that the brain processes information not only in *deterministic* terms but also through probability distributions that represent knowledge and uncertainty [66]. More specifically, it is well established that certain neural circuits are designed to manage uncertainty using probabilistic principles within a *Bayesian* framework, where perceived experience itself can shape prior states and beliefs [12]. This not only enables *Bayesian* inference but also raises the question of whether the uncertainty states observed in quantum domains might also be present in the brain’s molecular and synaptic structures. Indeed, Gentili [30] suggests that human cognition and its neural networks can be modeled using fuzzy logic and quantum probabilistic principles, aligning with the theory of quantum cognition originally proposed by Busemeyer [14]. The term quantum cognition refers to the idea that, while the origins of information processing may not be inherently quantum, certain mathematical structures allow cognitive phenomena to be accurately predicted using quantum probabilistic models [13]. This innovative framework has garnered support from recent studies indicating that quantum mathematics can address unpredictability in certain cognitive states, particularly those related to decision-making and information processing [65]. Previous lines of research align with the computational framework of *QBism*, which posits that quantum mechanics does not establish a new ontology but should instead be viewed as a mathematical tool for modeling complex phenomena that cannot be captured by Newtonian mechanics. Conscious experience may be one such phenomenon [28].

Within the domain of conscious experience, McFadden [54] proposed an alternative perspective aligned with quantum biology, linking the emergence of conscious experience to electromagnetic fields interacting within neural networks. These electromagnetic interactions and the brain’s electrochemical communications are hypothesized to exhibit properties that can be modeled and predicted using quantum mathematics [53]. Another prominent theory, rooted in the work of Hameroff and Penrose [35], suggests that conscious experience and levels of awareness can be predicted through molecular changes in cellular microtubules. According to this hypothesis, these molecular changes follow quantum rules, rendering them predictable within a quantum framework. Although these theories are conceptually well-grounded, they remain speculative due to (a) insufficient empirical research to conclusively support any single model and (b) their incomplete treatment of the multifaceted and subjective nature of conscious experience.

In the realm of anomalous cognition, quantum-component theories have been widely debated [79]. Among the earliest proposals is von Lucadou’s [78] *model of pragmatic information* (MPI), which conceptualizes anomalous cognitions, such as precognition, as operating through mechanisms analogous to quantum nonlocality. The MPI introduces the principle of non-transfer, offering an explanation for the inherent difficulty in replicating anomalous cognition under controlled laboratory conditions [31]. This model, grounded in indeterminism, complements more intricate theoretical constructs, such as the generalized quantum theory developed by Walach and von Stillfried [80]; see also Atmanspacher et al. [4].

### 1.3 The *Nonlocal Plasticity Theory* (NPT) and the *Guppy Effect*

Osherson and Smith [62] developed prototype theory, which led to the identification of the *Guppy Effect*. This effect suggests that a conceptual response is more likely to align with two related prototypes when presented simultaneously rather than separately. For example, given prototype 1 (*dog*) and prototype 2 (*domestic*), the concept *pet* (conceptual response) aligns more strongly with both prototypes when they are presented together. Building on this probabilistic logic, Aerts and Sozzo [3] argued that coinciding prototypes create an entanglement between them, resulting in the most fitting conceptual response [2], [1]. This work marked the first attempt to apply quantum mathematics, specifically Bell’s inequality violations [9], [17], to predict phenomena beyond strictly quantum systems. The *Guppy Effect* plays a pivotal role in explaining nonlocal entanglement effects within the *Nonlocal Plasticity Theory* (NPT). NPT also provides a framework for understanding the sources, mechanisms, and processes underlying anomalous cognitions.

Developed by Escolà-Gascón [24], NPT seeks to explain how anomalous cognitive information flows occur in relation to the environment. The theory is grounded in three core principles and their corresponding ontological postulates:

(1) *Principle of internity:* anomalous signals do not travel from the external environment to the cognitive system. Instead, they emerge from meaning systems embedded within experience (e.g., prototypes and concepts) and are associated with decision-making processes.
(2) *Principle of unconsciousness:* the processing of anomalous information occurs unconsciously and without deliberate intervention. If intervention takes place, it manifests in the conscious state rather than during unconscious processing. This unconscious processing produces an individual sensation of “knowing something” without understanding how this knowledge was acquired.
(3) *Principle of nonlocality:* anomalous information operates beyond the linear space-time continuum, behaving nonlocally. To evaluate and apply these principles, NPT proposes leveraging biological mechanisms involved in implicit learning and neuroplasticity.

Implicit learning shapes the attribution of meaning in decision-making, ensuring unconscious information processing. Rooted in reflex conditioning, NPT suggests that implicit learning can break the locality of stimulus contingencies. Experimentally, this can be achieved by disrupting the simultaneity required in reflex contingencies. While operant conditioning allows temporal distance between contingencies, reflex conditioning relies on local interactions (specific in space and time). Violating locality in reflex conditioning introduces a mechanism analogous to nonlocality. Escolà-Gascón [24] referred to this form of reflexive and implicit learning as quantum-like learning*.* If this concept were flawed, experiments would not have observed the accelerated learning curves reported by Escolà-Gascón [24]. Such results, observed under quantum-analogous conditions, justify further empirical investigation into which quantum properties may or may not apply to quantum-like learning.

Neuroplasticity, meanwhile, draws on the concept of translocalization [36]. Translocalization describes synaptic morphological changes that do not follow deterministic cause-effect sequences and instead occur (or collapse) far from the initially designated brain regions responsible for specific functions. This translocalized plasticity operates with high uncertainty levels, consistent with quantum nonlocality [37]. Based on this, NPT proposes a molecular biological marker to determine whether nonlocal plastic changes occur. Consequently, nonlocality can be tested on two levels: cognitive (based on implicit learning) and neurological (based on translocalized plasticity).

The *Guppy Effect* and the entanglement hypothesis proposed by Aerts and Sozzo [3] can be empirically integrated into and tested through NPT. Escolà-Gascón’s [24] experimental design introduces contingencies that violate locality, inherent in classical implicit learning, by isolating them. If these contingencies act nonlocally, the observed significant learning curve which theoretically should not occur can be explained through cognitive entanglement as proposed in the *Guppy Effect*. Participants' responses to nonlocal contingencies align probabilistically due to entanglement. This hypothesis of cognitive entanglement has been supported by probabilistic theoretical demonstrations in several studies [32], [34], [7]. This research aims to explore the extent of the hypothetical entanglement effect in the *Guppy Effect* as applied to quantum-like learning.

### 1.4 Objectives and hypotheses

Based on the above, the following four hypotheses are proposed:

(1) Greater predisposition to neuroplasticity through angiogenesis is associated with higher quantum-like learning performance levels.
(2) Quantum-like learning responses of monozygotic twins and their mental states measured via electroencephalography are more highly correlated under entanglement conditions than in non-entanglement experimental conditions.
(3) The *Guppy Effect* in quantum-like learning, if it occurs, will produce systematic increases in correct responses during quantum-like learning tasks.
(4) A factorial coefficient can integrate nonlocal correlations from Bell’s inequality with the variance explained by participants' response patterns in quantum-like learning. Therefore, this research combines theoretical foundations, empirical experimentation on the biology of consciousness and anomalous cognition, and data analysis using a novel statistical procedure that integrates quantum and classical correlations to address the hard problem of consciousness through an implicit learning-based approach.

## 2 Methods

### 2.1 Minimum required sample size

The required sample size for our experiment was estimated using the statistical criteria outlined by Escolà-Gascón [23], based on the distributions of the φ coefficient employed to evaluate contrast power. A standardized effect size of 0.5 was specified, applying the equations from page 373 of the cited manual, specifically Eq. (23). With a power level set at 0.99, the analysis indicated that the required sample size would range between 50 and 60 participants. Consequently, each group needed a minimum of 50 participants to ensure the detection of moderate and statistically significant effects in hypothesis testing.

### 2.2 Sample description

The final sample consisted of 106 pairs of monozygotic twins (*N* total = 212) who had been raised in the same familial environment for at least 15 years (mean age = 39.40; standard deviation = 3.86). While there were technically 212 participants, each twin was paired with their genetically identical sibling. This approach allowed us to utilize statistical analyses tailored for related samples. All participants provided informed consent to participate in the study. Their involvement was both voluntary and anonymous, with no financial incentives offered. No incidents were reported during data collection, and monozygotic twin status was medically confirmed for all cases through genotype testing which had been conducted before this study and which was available in the database we used to access the sample. The decision to work with monozygotic twins was based on prior evidence suggesting a form of nonlocal synchrony between identical twins, analogous to potential quantum entanglement effects [76]. If this hypothesis were valid, studying twins would provide a methodological advantage by enhancing the detectability of the targeted effects. Thus, this approach was chosen for statistical optimization and in accordance with previously published recommendations within our line of research. Likewise, the study design was also reviewed and approved by the regional ethics committee.

The sample was recruited using a medical database shared by several hospitals in Spain for scientific research purposes. Initially, 243 pairs of twins were contacted to assess their willingness to participate—of these, 137 expressed interest in the study. Due to logistical constraints, however, only 106 pairs ultimately completed the experiments.

### 2.3 Allocation of twin pairs to experimental groups

Each pair of twins was randomly assigned to one of two critical experimental conditions. The participants of the first group (group 0, comprising 53 twin pairs, totaling 106 participants) completed 144 implicit learning trials under nonlocality conditions without quantum entanglement. In the second group (group 1, also comprising 53 twin pairs), trials were conducted under nonlocality conditions with quantum entanglement, as detailed in subsection 2.7. Each trial featured four types of stimuli. Two were explicit stimuli: one associated with the biophysical technique of *continuous flash suppression* (CFS) and the other consisting of 60 points exhibiting random motion, generated using the *random dot motion* (RDM) technique. The remaining two were entirely concealed stimuli, imperceptible within the classical framework of conscious sensory experience. One concealed stimulus was emotional (positive or negative), while the other involved a uniform motion of 60 points, moving exclusively to the left or right. In this setup, participants were required to use only the explicit stimuli to anticipate the uniform motion direction of the 60 points, which initially moved randomly. Participant responses were recorded using the left (←) and right (→) arrow keys on a keyboard.

### 2.4 Control methods employed and stimulus characteristics

The 144 trials followed an implicit learning paradigm, measured under specific biophysical and empirical conditions described below. Some of these conditions are detailed extensively in the published report by Escolà-Gascón [24] and in the original proposal by Lufityanto et al. [51].

First, the RDM technique involves visually presenting 60 moving dots within a circular frame of varying diameters. The motion of these dots is defined by coherence levels, expressed as percentages. Higher coherence indicates greater uniformity in the dots' motion. In our design, two types of RDM stimuli were used: the explicit RDM stimulus and the hidden RDM stimulus. The explicit or perceptible RDM stimulus was projected for 400 microseconds and consisted of completely random motion of the 60 dots. Since the motion was entirely random, the coherence level was absolute zero, ensuring participants could not use analytical or deductive processes to anticipate the uniform motion of the dots. In contrast, the hidden RDM stimulus involved the systematic and uniform motion of the 60 dots in a single direction (left or right). This hidden RDM stimulus was never directly displayed to participants, but our circuits were programmed to ensure its occurrence.

Second, the hidden emotional stimuli consisted of images with varying levels of valence and arousal, calibrated using the *Geneva Affective Picture Database* (GAPED) [20]. The images were either positive (eliciting pleasant states of relaxation) or negative (containing aversive or overstimulating content inducing high levels of stress or arousal). From over 400 photographs, 18 images were selected for their most polarized valence values: the lowest values corresponding to relaxation and the highest values associated with anxiety or stress. A critical aspect of the experiment was that each trial included an emotional image (positive or negative) contingently associated with a specific uniform motion (left or right) of the hidden RDM stimulus. When the dots moved left, the associated emotional image was negative; when the dots moved right, the associated image was positive. Crucially, neither the emotional images nor the uniform motion of the dots were accessible to participants' perception, preventing logical, analytical, or deductive reasoning to anticipate the motion. Emotional images were adjusted to a specific sepia tone, creating a visual distortion that ensured they remained imperceptible.

Finally, the CFS technique was applied consistently across all 144 trials. This technique involves the progressive and sustained projection of geometric light shapes in multiple colors (excluding sepia). These light shapes, or flashes, were configured to vary in opacity. Under the strictest nonlocality conditions, this stimulus was rendered completely opaque and superimposed over the hidden emotional stimulus, effectively obscuring the emotional image and making it visually imperceptible to participants. The stimuli were presented using a stereoscope and a chin rest that immobilized participants' heads. The stereoscope was synchronized with the stimulus projections on a 20-inch computer screen, and the chin rest ensured a fixed distance of 57 centimeters between the participants and the monitor. These details are critical, as the synchronization between the stereoscope, monitor, and chin rest is essential for systematically reproducing this protocol in future studies.

### 2.5 Criteria for stimulus design and trial sequencing

**Fig. 1:**
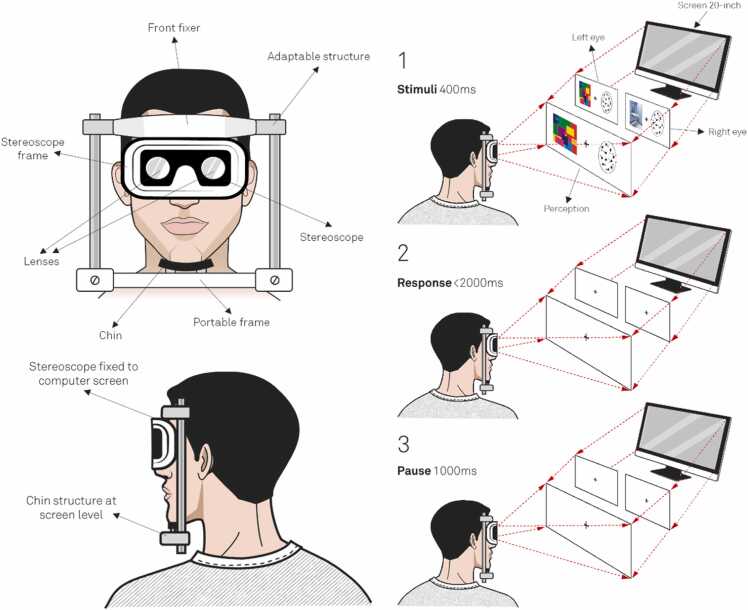
**Sequence of steps for each experimental trial.** Visualization of the steps and actions performed in each trial, including the projection of stimuli through the stereoscope and the specifications of the chin rest and its design. In screens where no stimuli were presented and a plus symbol (+) appeared, there was no stimulus exposure. In the final phase (phase three), the hidden left-right RDM stimulus was introduced. This process was repeated 144 times for each participant. **Notice:** This figure, along with some of its illustrations or parts, was previously used in the Open Access publication by Escolà-Gascón [24]. The author of this report retains full reproduction rights and permission to reuse them in this article.

At this point, we would like to outline the essential criterion used to establish the synchronizations described earlier. To ensure the intended illusory effect and effectively conceal the respective stimuli, it was necessary to determine each participant’s hemispheric dominance. For right-handed participants, the explicit RDM stimulus was projected to the right eye, while the CFS stimulus overlapping the emotional image was presented to the left eye. Conversely, for left-handed participants, the explicit RDM stimulus was projected to the left eye, with the CFS stimulus displayed to the right eye. To provide a clear overview of the stimulus configuration and trial setup, we refer to Fig. 1.

Each trial consisted of three distinct phases. In the first phase, the stimuli were projected as outlined in Fig. 1. This projection lasted 400 microseconds. During this phase, the explicit RDM stimulus—comprising completely random motion of the points—was presented to either the right or left eye, depending on the participant’s hemispheric dominance. As depicted in Fig. 1, for right-handed participants, the CFS stimulus was displayed exclusively in the left eye, while the random-motion RDM stimulus was presented in the right eye.

In the second phase, a blank screen was displayed. During this interval, participants used the left or right arrow keys on a keyboard to predict the uniform motion of the points. This response phase had a maximum duration of 2000 microseconds. Once the participant provided their response, the trial advanced to the third phase, during which the uniform motion of the points was displayed. If the participant’s response matched the direction of the uniform motion, it was recorded as a correct response (1) in the response matrix. If the response did not match, it was recorded as an error (0).

It is important to note that the concealed emotional stimulus associated with the binary motion of the points was presented during the first phase of each trial. Implicit learning performance was evaluated by summing the number of correct responses at the end of each sequence.

### 2.6 Experimental conditions of nonlocality

This biophysical experiment involved nonlocality conditions that violated three fundamental principles of classical learning (reflex conditioning) both empirically and biologically. These principles, typically required to ensure effective learning, are as follows:

(1) *Principle of local simultaneity.* The contingency between associated stimuli must occur within the same space-time continuum; greater temporal and spatial discrepancies reduce the likelihood of successful learning. For example, in ethology, when training police dogs to recognize specific odors (e.g., illegal substances), these odors must be associated with specific locations (e.g., a hidden compartment in a suitcase). During training, the concealed exposure of the “illegal substance” stimulus must coincide with the “hidden compartment” location. If this alignment is disrupted (e.g., the substance is placed elsewhere), the association weakens. To address this, trainers systematically repeat the pairing of substance *X* with compartment *Y* to enhance the dog’s learning efficiency. In this study, the simultaneity and sensory accessibility of stimulus associations were completely blocked by superimposing the CFS stimulus over the emotional image, which remained hidden behind the CFS stimulus. This setup intentionally disrupted local simultaneity.
(2) *Principle of temporal coherence.* In instrumental learning (operant conditioning), temporal coherence between the target behavior and the reinforcing reward is essential. This principle dictates that a reward should ideally be delivered immediately after the desired behavior is performed. As the temporal gap between the behavior and reinforcement increases, the likelihood of repeating the behavior decreases, necessitating more contingencies to achieve the desired learning outcome. Conversely, in classical conditioning, the reward can act as an antecedent (unconditioned stimulus associated with a neutral stimulus) to elicit a specific behavior, as seen in Pavlov’s experiments where dogs salivated in response to a bell sound associated with food. Temporal coherence dictates that delays between the bell and the delivery of food diminish the efficiency of learning. In our experiment, this principle was deliberately violated because the uniform motion of the points in the hidden RDM stimulus occurred only after, and not before, participants provided their responses. The only concealed stimulus presented during the initial phase was the emotional image. Consequently, our protocol deviated from this logical system, creating conditions analogous to nonlocality.
(3) *Principle of collinearity*. This principle concerns the consistency of contingencies, particularly in the motion of points within the RDM stimulus. Mathematical collinearity posits that as the consistency of random motion increases, the motion becomes more predictable. Researchers typically assess adherence to this principle by analyzing the linear relationship between consistency levels and correct responses per trial (e.g., summing the number of “1” values in each column of the response matrix [51]). For instance, a consistency level of 10 % implies that 6 out of 60 points exhibit uniform motion, making the motion no longer purely random. In our study, however, consistency levels were set to zero, and random sequences were generated using *IBM Brisbane’s* quantum supercomputer to ensure pure randomness. Consequently, no linear correlation between consistency levels and the probability of correct responses was expected. This deliberate violation of the principle supported the presence of nonlocality in the implicit learning examined.

**Fig. 2:**
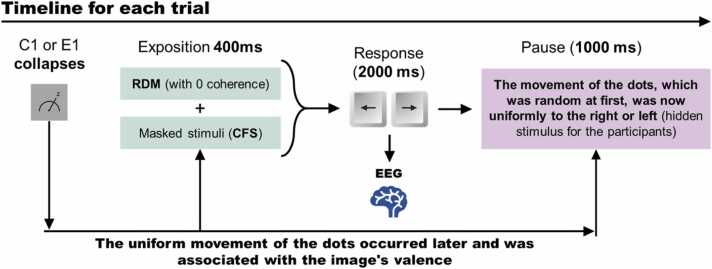
**Steps for developing the treatment-intervention for the experimental group.** This diagram illustrates the steps taken for each trial to facilitate the reproducibility of the experiment. The figure shows that random events generated by *IBM Brisbane* are emitted asynchronously with the presentation of the hidden RDM stimulus involving uniform point movement.

Fig. 2 provides a logical schematic illustrating these violations of fundamental principles in the ethology and biology of learning. Notably, binary measurements were recorded simultaneously at the start of the experiment, enabling the assignment of positive or negative images during the initial projection phase of each trial.

### 2.7 Quantum entanglement configuration

#### 2.7.1 Generation of collapses in concealed uniform RDM movements

The study involved 106 pairs of monozygotic twins, with 53 pairs assigned to group 0 (control) and 53 pairs to group 1 (experimental). These pairs were randomly distributed across two quantum conditions that determined the configuration and structure of the binary uniform RDM movements (represented as 0 and 1). The *IBM Brisbane* quantum supercomputer was utilized to implement two distinct circuits, each featuring two qubits (labeled *q*_*0*_ and *q*_*1*_). For readers unfamiliar with certain quantum computing concepts, Appendix A of this report offers basic definitions and explanations of key terms mentioned throughout.

The first circuit, known as the *control circuit* (C1), contained two *Hadamard* gates (*H*), with no quantum entanglement between the qubits. The *H* gates enabled the qubits to operate in a state of superposition, ensuring maximum uncertainty and randomization. In the absence of entanglement, the values in the density matrix were not expected to violate Bell’s inequality, and nonlocal correlations were anticipated to be minimal or non-significant. Binary measurements, or collapses into 0 or 1, were generated via the wavefunction. Due to the maximal uncertainty induced by the *H* gates, the sequences of collapses were strictly random. Given the two qubits, two types of matrices (A and B) were generated, each with dimensions of 53 × 144. The total 53 + 53 = 106 corresponds to the number of twin pairs in the study, while the 144 columns represent the 144 trials conducted in the experiment. Matrix A was used for the first block of paired twins (group 0-A and group 1-A), while matrix B was assigned to the remaining twins (group 0-B and group 1-B).

**Fig. 3:**
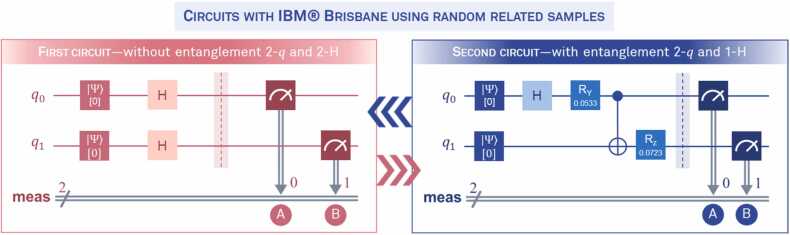
**Specifications of the two designed circuits.** Quantum circuits implemented on the *IBM Brisbane* supercomputer for generating binary collapses from quantumly independent and entangled qubits. This study examines the impact of partial entanglement induced by randomized noise gates on the efficiency of learning sequences under empirical nonlocality conditions. The first circuit is designated as C1 (control), while the second is labeled E1 (experimental).

Out of the 106 twin pairs, 53 performed the experiment using C1, which lacked qubit entanglement. The remaining 53 pairs were randomly assigned to the second circuit, referred to as the *experimental circuit* (E1). In E1, a single *H* gate was applied to *q*_*0*_, followed by the introduction of a Bell state on *q*_*1*_, which connected it to the states of *q*_*0*_. The Bell state was implemented using CNOT algorithms, which create perfect entanglement. To simulate realistic conditions, acknowledging that all physical systems inherently involve some degree of noise, two additional gates (*R*_*y*_ and *R*_*z*_) were introduced to add random perturbations to *q*_*0*_ and *q*_*1*_. Noise (*R*_*y*_) was applied to *q*_*0*_ prior to the CNOT gate, which produced the Bell inequality violation, while noise (*R*_*z*_) was applied to *q*_*1*_ after the CNOT gate. Both C1 and E1 generated binary collapses. In C1 the collapses were entirely randomized; in E1 the collapses were also random but influenced by the entanglement between the qubits. This design enabled the contrast and analysis of entanglement effects within the framework of implicit learning models, as explored in this study. Fig. 3 provides a schematic representation of the logical configurations of these circuits.

#### 2.7.2 Mathematical demonstrations

The circuits we designed rely on algorithms that are straightforward to implement in quantum computing. For circuit C1, the operation of the *Hadamard* gate for *q*_*0*_ (∣0〉) and *q*_*1*_ (∣1〉) is represented in (1), (2):

(1) $$H|0\rangle \;\;=\frac{|0\rangle \;\;+|1\rangle \;\;}{\sqrt{2}}$$

and

(2) $$H|1\rangle \;\;=\frac{|0\rangle \;\;-|1\rangle \;\;}{\sqrt{2}}$$

Consequently, after applying *H* to both qubits initialized with ∣00〉, the outcome is as described in (3), (4):

(3) $$|00\rangle \;\;\overset{H\left(0\right)}{⟶}=\frac{|0\rangle \;\;+|1\rangle \;\;}{\sqrt{2}}⊗|0\rangle \;\;=\frac{|00\rangle \;\;+|10\rangle \;\;}{\sqrt{2}}$$

and

(4) $$|00\rangle \overset{H\left(1\right)}{⟶}=\frac{1\;\;}{\sqrt{2}}\left(|0\rangle +|1\rangle \right)⊗\frac{|0\rangle \;\;+|1\rangle \;\;}{\sqrt{2}}=\frac{1}{2}\left(|00\rangle +|01\rangle +|10\rangle +|11\rangle \right)$$

⊗ denotes the tensor product. Since the final state of circuit C1 is a maximally mixed state with equal probabilities for all binary combinations, the collapses in matrices A and B, each with dimensions of 53 × 144, should also exhibit random structures. If matrices A and B are random, they must also be independent of each other. This implies that when these structures are used in configuring the contingencies of the stimuli, as described in subsections 2.4 and 2.5, the keyboard responses of participants in matrix A should not predict the structure of matrix B, and vice versa. Circuit E1 is more complex but also offers mathematically straightforward formulations, starting with the initial *Hadamard* gate applied to *q*_*0*_ in Eq. (5):

(5) $$|00\rangle \;\;\overset{H\left(0\right)}{⟶}=\frac{1}{\sqrt{2}}\left(|00\rangle \;+|10\rangle \;\right)$$

The *R*_*y*_(θ) gate introduces a random rotation angle along the *y*-axis. Its effects on the state represented in Eq. (5) are described in Eq. (6):

(6) $$\begin{array}{l} R_{y}\left(\theta_{q}0\right)\frac{1}{\sqrt{2}}\left(\left|00\right\rangle \;+\left|10\right\rangle \;\right)\Rightarrow \frac{1}{\sqrt{2}}\;\;\times \\ (\cos\left(\theta_{q}0/2\right)\left|00\right\rangle \;+\;\sin\left(\theta_{q}0/2\right)\left|01\right\rangle +\\ \cos\left(\theta_{q}0/2\right)\left|10\right\rangle \;+\;\sin\left(\theta_{q}0/2\right)\left|11\right\rangle ) \end{array}$$

Next, a CNOT (*CX*) gate is introduced, applying the necessary conditions to generate a Bell state, with *q*_*0*_ serving as the control and *q*_*1*_ as the target. While the CNOT gate is not itself a Bell state, its role is pivotal, as it establishes nonlocal correlations between the qubits, increasing the likelihood that the density matrix of the states will violate Bell’s inequality. The effects of the *CX* gate are expressed in Eq. (7):

(7) $$\begin{array}{l} CX\frac{1}{\sqrt{2}}\;\;\times \\ (\cos\left(\theta_{q}0/2\right)\left|00\right\rangle \;+\;\sin\left(\theta_{q}0/2\right)\left|01\right\rangle +\\ \cos\left(\theta_{q}0/2\right)\left|10\right\rangle \;+\;\sin\left(\theta_{q}0/2\right)\left|11\rangle \right)\Rightarrow \\ \frac{1}{\sqrt{2}}\;\;\times \\ (\cos\left(\theta_{q}0/2\right)\left|00\right\rangle \;+\;\sin\left(\theta_{q}0/2\right)\left|01\right\rangle +\\ \cos\left(\theta_{q}0/2\right)\left|11\right\rangle \;+\;\sin\left(\theta_{q}0/2\right)\left|10\rangle \right) \end{array}$$

A second gate, *R*_*z*_(θ), was also introduced on *q*_*1*_, immediately after the CNOT, applying another random rotation—this time along the *z*-axis. The resulting effects are presented in Eq. (8):

(8) $$\begin{array}{l} R_{z}\left(\theta_{q}1\right)\frac{1}{\sqrt{2}}\;\;\times \\ (\cos\left(\theta_{q}0/2\right)\left|00\right\rangle \;+\;\sin\left(\theta_{q}0/2\right)\left|01\right\rangle +\\ \cos\left(\theta_{q}0/2\right)\left|11\right\rangle \;+\;\sin\left(\theta_{q}0/2\right)\left|10\rangle \right)\Rightarrow \\ \begin{array}{l} \frac{1}{\sqrt{2}}\;\;\times \\ (\cos\left(\theta_{q}0/2\right)e^{i\theta_{q}1/2}\left|00\right\rangle +\;\sin\left(\theta_{q}0/2\right)e^{i\theta_{q}1/2}\left|01\right\rangle +\\ \cos\left(\theta_{q}0/2\right)e^{i\theta_{q}1/2}\left|11\right\rangle +\;\sin\left(\theta_{q}0/2\right)e^{i\theta_{q}1/2}\left|10\right\rangle ) \end{array}\\ \;\\ \; \end{array}$$

The rotations adjust the amplitudes of the basis states (both before and after the CNOT), creating entanglement while ensuring that the collapses in matrices A and B are not identical. Both C1 and E1 generated two matrices, A and B, containing collapses; however, in E1, the binary values in matrices A and B were derived from entangled qubits. This study aims to explore whether configuring the contingencies of stimulus exposure in an implicit learning experiment using entangled qubits could impact or disrupt the efficiency of participants' success rates.

#### 2.7.3 The *Quantum-Multilinear Integrated Coefficient* (Q)

The aim of this subsection is to introduce a new set of equations to derive a complex correlation coefficient that integrates nonlocal correlations (from quantum statistics) with local correlations (multilinear correlations from classical statistics). This new statistical measure is termed the *Quantum-Multilinear Integrated Coefficient* (referred to hereafter as the *Q* coefficient). The formulation of this new coefficient is based on the hypothesis that certain aspects of conscious experience exhibit nonlocality, which could influence organismal behavior. Specifically, we propose that nonlocality affects the factors that condition—without establishing causality—the configuration of stimulus contingencies in our experiment, which, in turn, shape participants’ decision-making processes.

In our experimental design, these conditioning factors arise from the probabilistic states of the qubits. From these qubit states, the density matrix is derived, which forms the basis for analyzing nonlocal correlations, violations of Bell’s inequality, and entanglement. If quantum information flows from the entangled qubits to the collapse of the wavefunction, a connection between quantum entanglement and participant efficiency (i.e., success/error rates) can be expected. Participant efficiency is represented by decision states (1 = success, 0 = failure) that are determined by matches between the A-B collapse matrices and individual keyboard responses. When a keyboard response (left or right) aligns with the uniform motion of the points (left or right), it is recorded as a success. If no alignment occurs, it is recorded as a failure.

To investigate whether qubit entanglement subtly impacts the collapses, the first step is to verify the violation of Bell’s inequality and calculate nonlocal correlations using the qubits’ density matrix. After the final rotation, the state of the qubits exists as a complex superposition, dependent on the values of θ*q*_*0*_ and θ*q*_*1*_, with the initial density matrix expressed as (see Eq. (9)):

(9) $$\rho_{0}=\left|\psi \right\rangle \left\langle \psi \right|$$

With partial depolarization (see Eq. (8)), the density matrix is adjusted according to Eq. (10):

(10) $$\rho \prime =\left(1-p\right)\rho_{0}+\frac{p}{4}I$$

The density matrix is a 4 × 4 matrix, and its values and structure will be detailed in the results subsection, based on the application of the circuits to the 106 participants paired across groups (53 pairs receiving C1 and 53 receiving E1). At this stage, we present the equations necessary to calculate the nonlocal correlations and the *Bell parameter* (*S*), measured using the *Clauser-Horne-Shimony-Holt* (CHSH) criterion. Subsequently, we will introduce the equations pertaining to multilinear correlations.

*Quantum correlations* (*O*) are derived from the trace of the adjusted density matrix, as represented in Eq. (11):

(11) $$\mathrm{Correlation}\left(O\right)=\mathrm{Tr}\left(\rho \prime \times O\right)$$

where

(12) $$O=C_{ij}=\left\langle \sigma_{i}⊗\sigma_{j}\right\rangle$$

σ_*i*_ and σ_*j*_ are *Pauli* operators corresponding to the bases *i* and *j*. According to our circuit, we have the following nonlocal correlations (Eq. (13)):

(13) $$\begin{array}{l} C_{XX}=\mathrm{Tr}\left(\rho \prime \left(\sigma_{X}⊗\sigma_{X}\right)\right)\\ C_{YY}=\mathrm{Tr}\left(\rho \prime \left(\sigma_{Y}⊗\sigma_{Y}\right)\right)\\ C_{ZZ}=\mathrm{Tr}\left(\rho \prime \left(\sigma_{Z}⊗\sigma_{Z}\right)\right) \end{array}$$

Once the nonlocal correlations are obtained, the *S* value (or Bell’s *S*) can be calculated using the CHSH criterion, as shown in Eq. (14):

(14) $$S=\left|C_{XX}-C_{YY}+C_{ZZ}\right|$$

In addition to the nonlocal correlations and Bell’s *S* value, we analyzed the degree of concurrence to ensure entanglement was achieved, using (15), (16). The calculation of *concurrence* (Con) is performed using Eq. (15):

(15) $$\rho **=\left(\sigma_{i}⊗\sigma_{j}\right){\rho \prime }^{*}\left(\sigma_{i}⊗\sigma_{j}\right)$$

Building on the above, the calculation proceeds with Eq. (16):

(16) $$\mathrm{Con}=\;\max\left(0.2\lambda_{\max}-\sum \lambda_{i}\right)$$

where, λ_*i*_ are the autovalues of ρ’ and ρ* *.

To meet Bell’s inequalities, the criteria require *S* > 2, Con > 0, and nonlocal correlations > 0. These thresholds are standard for circuits of this type [16], [11], [45].

Once the nonlocal correlations, *S* value, and concurrence are calculated, the multilinear correlation matrix must be derived from the participants' keyboard responses using the arrow keys (← →). The logic is as follows: both C1 and E1 circuits produced binary collapses that generated matrices A and B. However, unlike C1, the collapses in E1 originated from entangled qubits. In the twins subjected to C1 *and* the twins subjected to E1, the A and B matrices of collapses were used to configure the contingencies linking emotional stimuli to the uniform motion of the points. During each trial, participants responded by pressing one of the keyboard arrows (left or right) to predict the uniform future motion of the RDM points.

In classical learning processes, contingencies are localized in space and time, ideally occurring under simultaneous conditions. However, in the S144 sequence of Escolà-Gascón’s [24] experiment, the uniform motion of the RDM points only occurred after participants made their response, making it a future stimulus. This setup violated the locality principle required for classical implicit learning (but not for quantum learning). Escolà-Gascón [24] observed that despite this violation, a significant learning curve emerged, leading to the term quantum-like learning, hypothesizing that nonlocal mechanisms might influence the process. The present study seeks to test whether quantum entanglement had any measurable effect.

In Escolà-Gascón’s [24] protocol, the only stimulus presented before participants’ responses was the emotional images. While the random 0 or 1 collapses had already occurred, no uniform motion had been assigned to the RDM points at the start of each trial. Thus, participants could neither know nor predict how these collapses would later align with the uniform motion of the RDM points. This is critical because the initial RDM motion was entirely random (see subsection 2.5). Based on this setup, we hypothesize that if entanglement influences quantum-like learning, its effects should manifest in the participants' reaction matrices (← →) rather than in the collapse matrices. Consequently, the *tetracoric* correlation matrix—appropriate for binary responses—should be calculated using the keyboard response matrices.

The primary objective of analyzing the *tetracoric* matrix is to identify systematic patterns or structures that distinguish between entangled and non-entangled conditions. This is achievable through factorization, where eigenvalues (λ) are used to detect stable, non-random patterns in the matrix (commonly referred to as latent variables), and the variance explained by these eigenvalues (λ²) is calculated.

A critical statistical consideration is determining how many eigenvalues or latent variables to retain to identify systematic variance rather than random noise. While several criteria exist, with Kaiser’s [43] criterion being the most widely used, we recommend a more sensitive and effective method for binary matrices: parallel analysis applied to a scree plot [70]. This method compares observed eigenvalues, ranked from largest to smallest for each trial, with simulated eigenvalues generated under ideal random conditions. Visually, this generates a sediment curve, and the goal is to retain eigenvalues up to the point where the observed and simulated curves intersect [50]. The explained variance can then be calculated by dividing the total sum of the retained eigenvalues by the total number of trials (144 in this experiment). For nonlocal correlations, Bell’s *S* value serves as a combined indicator of these correlations (see Eq. (14)).

With this foundation, we propose the fundamental Eq. (17) for the *Q* coefficient:

(17) $$Q=V_{k}\cdot \left(1+\beta \cdot C_{q}\cdot S\right)$$

where:

*V*_*k*_ represents the explained variance associated with the observed eigenvalues in the *tetracoric* correlation matrix of the experimental group;

*C*_*q*_ denotes quantum concurrence (see Eq. (16));

*S* is the combined value of nonlocal correlations calculated using the CHSH criterion (see Eq. (14));

β is the estimation parameter used to derive *Q*. This parameter calibrates the metric of the product *C*_*q*_ *⋅ S*, ensuring that smaller entanglement effects correspond to smaller β values, and vice versa.

Determining the appropriate β value is a nuanced statistical challenge. Ideally, it could be estimated using a dataset similar to ours, by employing a function that minimizes the differences between the explained variance of the experimental group and that of the control group. This calculation would be based on the success/error matrix rather than the keyboard response matrix, for which the explained variances (*V*_*k*_) are already known. This approach can be formalized as shown in Eq. (18):

(18) $$\min_{\beta }{\sum_{i}\left(Q_{\mathrm{Observed}}-Q_{\mathrm{Calculated}}\right)}^{2}$$

However, since we do not have additional sample sets and this is the first application of the *Q* coefficient, we must rely on an estimator based on explained variance that reflects how the experimental conditions of entanglement influence performance levels. There are several approaches to calculate this effect. In the context of our study, which involves control and experimental groups, changes in the variability of success/error rates can be analyzed using a 2 × 2 multifactorial ANOVA model, with partial eta-squared as the chosen statistic. Therefore, we propose that the estimation of β be represented by this statistic, as it serves as a measure of explained variance.

To refine this approach, we will specifically use the explained variance corresponding to the fixed effects of the interaction, accounting for the distinction between entangled and non-entangled conditions, as well as the impact of twin pairing. Interestingly, Fisher’s [26], [27] original method eliminates the need for iterative calculations, as his statistical inference framework was built upon analyzing squared differences—a foundation that led to the derivation and standardization of the *F* probability distribution [18], [25].

### 2.8 Biomarkers

#### 2.8.1 Electroencephalography (EEG) derived from Fourier transform

**Fig. 4:**
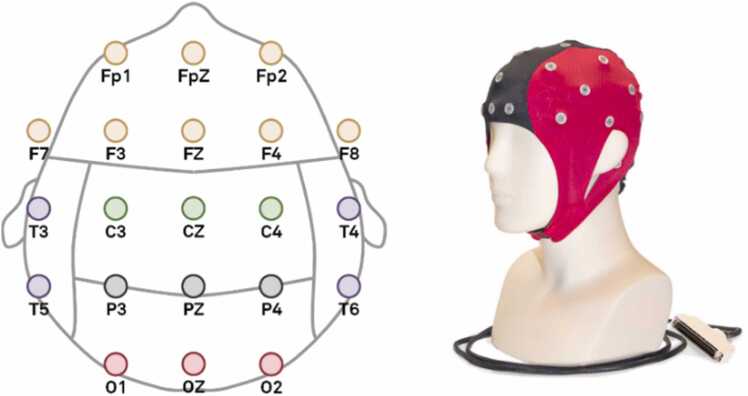
**Layout and configuration of the EEG channels.** Setup of the 21 channels organized by brain regions, following the international 10–20 system. The image on the right displays a photograph of the CS-356 EEG cap. **Notice:** This figure, along with some of its illustrations or parts, was previously used in the Open Access publication by Escolà-Gascón [24]. The author of this report retains full reproduction rights and permission to reuse them in this article.

The EEG device employed in this study was the *Waveguard Connect EEG Cap* (CS-356), configured with 21–32 channels following the international 10–20 system. Fig. 4 provides an illustration of this configuration and the electrode channels utilized. Brainwave measurements, which were used to define participants' mental states (specifically a working state in this experiment), were conducted using the *NeuroREC* software. This was implemented in collaboration with UNITY, which integrated 3D neuroimaging with topographic maps of the microvolt readings captured by each electrode-channel.

*NeuroREC* is designed to measure four distinct types of brainwaves based on their amplitudes: *delta* (1–4 Hz), *theta* (4–8 Hz), *alpha* (8–12 Hz), and *beta* (12–30 Hz). In this experiment, beta waves were associated with the working mental state. Frequency-domain analysis was conducted using the *Fast Fourier Transform* (FFT) to calculate the *power spectral density* (PSD, μV²/Hz) averaged per channel [19], [44].

The study included two main groups, each comprising 106 participants, with paired individuals in each group. EEG recordings and PSDs were analyzed for four subgroups (G0-A, G0-B, G1-A, G1-B), each containing 53 participants. The twins in G0-A and G0-B were paired, as were those in G1-A and G1-B. The implicit learning sequence S144, referred to as *psi* [24], had a maximum duration of 12 minutes, although the average execution time was typically shorter when completed without interruptions. For each participant, PSD values were recorded during the second immediately preceding their keyboard response (clicking either the left [←] or right [→] arrow keys). PSDs were averaged per channel for each participant, providing a summary of electrocortical activity throughout the experiment.

To identify the brain regions associated with neurophysiological working states, we focused on *regions of interest* (ROIs), classified according to the brain lobes delineated in Fig. 4. Guided by previous research, we concentrated primarily on the frontal and occipital ROIs, while also exploring potential variations across other lobes.

These measurements were included to test the hypothesis that neurophysiological states might predict the structure of collapses, which are attributed to entanglement effects. Successfully predicting or correlating collapse structures with EEG results would provide new evidence supporting the notion that quantum mechanics intersects with cognitive learning processes, an idea originally proposed by Busemeyer [14] and discussed in the introduction.

#### 2.8.2 Assessment of neuroplasticity, basal energy consumption, and cellular stress

According to Han et al. [37], the NPT postulates that certain morphological changes in the synapses of neural circuits occur translocally in individuals who demonstrate superior performance in implicit learning tasks. This translocalization of neuroplasticity arises through mechanisms that remain poorly understood. However, documented cases show that neural circuit reorganization often shifts away from regions genetically designated for specific functions—this phenomenon has been investigated in areas like language [48]. Building on this idea, the NPT proposes that such changes could potentially be predicted under the principle of uncertainty, aligning with models of nonlocal correlations in quantum mathematics.

Various mechanisms and factors contribute to neuroplasticity, one of the most significant being neurogenesis [42]. Studies have identified significant positive correlations between implicit learning performance and predisposition to neuroplasticity [61]. Moreover, medical interventions promoting neurogenesis have been shown to enhance specific cognitive abilities in patients [49]. These findings support the use of *Brain-Derived Neurotrophic Factor* (BDNF) as a biological marker. This marker is particularly valuable for evaluating the activity of proteins interacting with tyrosine kinase TrkB receptors, which regulate synaptic strength and intensity in the brain, thereby facilitating neuroplasticity and cellular regeneration [39]. The rationale for cellular regeneration is rooted in the preservation of critical cognitive functions necessary for survival, a concept closely tied to this study and the CBC model outlined in the introduction [29].

BDNF levels were measured using the *Rapid ELISA Kit* (CE marked, Avantor®), requiring only a single drop of blood. Measurements were taken at a single time point (normative values ranged from 10–30 ng/mL) before participants began the experiment. If individuals with a higher predisposition to neuroplasticity achieve more effective and efficient implicit learning, we expect to observe significant positive correlations between this test and their performance.

Since the mental states during participants’ keyboard responses (clicks on either the left [←] or right [→] arrow keys) were associated with working states that required sustained activation, we examined potential physiological correlations between performance, decentralized basal energy consumption, and stress levels. While working states measured via beta waves are not inherently linked to high energy consumption, the implicit learning conditions applied in this study were nonlocal and related to anomalous cognition. Identifying nonlocal quantum-like learning would necessitate finding positive and increasing correlations between physiological predispositions to energy consumption and participant accuracy.

Energy consumption was assessed by measuring *Free Fatty Acids* (FFA) using the Sigma Free *Fatty Acid Assay Kit* (MAK466, Merk®), with normative values ranging from 0.2–0.4 mmol/L. To evaluate physiological activation levels of the sympathetic nervous system, Alpha-Amylase levels were measured using the *Sigma α-Amylase Activity Assay Kit* (MAK478, Merk®), with standardized values between 20–90 U/L. Table 1 summarizes the devices used, the metrics applied, and the significance of each measurement.

**Table 1:** Summary of specific biomarkers: descriptions, reference ranges, and measurement characteristics.

**Biomarker**	**Range**	**Devices**	**Technique**	**Measurement**
***Brain-Derived Neurotrophic Factor*****(BDNF)**	10–30 ng/mL	*Brain-derived Neurotrophic Factor* (BDNF) Rapid™ ELISA Kit (CE marked), provided by Avantor®	ELISA (Enzyme-Linked Immunosorbent Assay)	Measures neurogenesis and plasticity; useful for capillary blood analysis.
***Free Fatty Acids*****(FFA)**	0.2–0.4 mmol/L	Sigma Free Fatty Acid Assay Kit, MAK466, provided by Merck®	Enzymatic colorimetric	Indicates metabolic flexibility and energy availability.
**Alpha-Amylase**	20–90 U/L	Sigma α-Amylase Activity Assay Kit, MAK478, provided by Merk®	Colorimetric/enzymatic detection	Reflects sympathetic activation and stress; suitable for capillary samples.

## 3 Results

### 3.1 Density matrices and nonlocal correlations

#### 3.1.1 Circuit without entanglement (C1)

The density matrix of the qubit states in the circuit with two *Hadamard* gates and no entanglement (C1) was as follows (see Eq. (19)):

(19) $$\rho \prime =\left\{\begin{array}{cccc} 0.25+0.j & 0.25+0.j & 0.25+0.j & 0.25+0.j\\ 0.25+0.j & 0.25+0.j & 0.25+0.j & 0.25+0.j\\ 0.25+0.j & 0.25+0.j & 0.25+0.j & 0.25+0.j\\ 0.25+0.j & 0.25+0.j & 0.25+0.j & 0.25+0.j \end{array}\right\}$$

The expression “0.*j*” denotes imaginary values in density matrix (see Eq. (19)). We have clarified this to avoid any confusion. The same interpretation should be applied to density matrix (20). The nonlocal correlations for Eq. (19) were as follows: *C*_*XX*_= 1.0000, *C*_*YY*_≈ 0, and *C*_*ZZ*_≈ 0. The Bell *S*-value following the CHSH criterion was 1.0000, and the concurrence indicator was 0. These results confirm that the qubits in this circuit were not entangled. The nonlocal correlations lacked sufficient magnitude, and the concurrence revealed that the eigenvalues of Eq. (19) did not satisfy the mathematical properties required for entanglement. This outcome was expected and based on the values of Eq. (19), we can determine that the qubits operated in a completely independent quantum state.

Given this information, the *Hadamard* gates in C1 were expected to produce entirely random binary collapses. To ensure that the C1 matrices A and B did not contain structures or patterns deviating from randomness, Shannon entropy indices were calculated. An entropy index of 1 or close to it indicates genuinely random sequences. The average entropies were 0.9948 and 0.9962, respectively, providing statistical evidence to support the randomness of these collapses. Additionally, the entropies for each vector were within the range > 0.9 < 1.

**Fig. 5:**
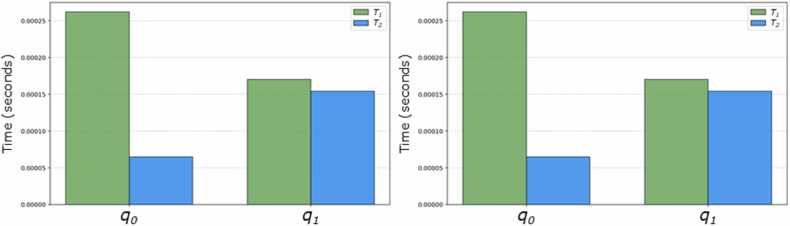
**Distributions of coherence times for each qubit in C1 (left) and E1 (right) circuits.** Coherence times were averages estimated from the *IBM Brisbane* supercomputer.

Since C1 was implemented on a physical quantum system using *IBM Brisbane’s* supercomputer, the reaction times of the qubits (*T*_*1*_ and *T*_*2*_) during circuit execution for each participant were also analyzed. This was achieved by accessing the backend of the supercomputer and using IBM’s password-protected API system. Fig. 5 includes two bar charts showing the average response times (in seconds) for both the C1 and E1 circuits.

The reaction times for C1 were exceptionally low. This indicated that the magnitude of disturbances potentially arising from these times was practically negligible, ensuring that errors resulting from such reactions did not significantly impact the quantum process of the applied circuit.

#### 3.1.2 Circuit with entanglement (E1)

The density matrix of the qubit states for circuit E1 is presented in Eq. (20):

(20) $$\rho \prime =\left\{\begin{array}{cccc} 0.4734+1.3936e^{-18}j & 0 & 0 & 0.4980-3.6082e^{-02}j\\ 0 & 0 & 0 & 0\\ 0 & 0 & 0 & 0\\ 0.4980+3.6082e^{-02}j & 0 & 0 & 0.5266-1.4303e^{-18}j \end{array}\right\}$$

The following nonlocal correlations were obtained for Eq. (20): *C*_*XX*_= 0.9960, *C*_*YY*_= -0.9960, and *C*_*ZZ*_= 1.0000. The Bell’s *S* value was 2.9920, indicating a clear violation of Bell’s inequality in this circuit and providing strong evidence that both qubits were properly entangled. The concurrence value, used to verify the entanglement of the qubits, was 0.8470, further confirming this condition. Additionally, the nonlocal correlations were significant, reinforcing the nonlocality of the qubits' states. The reaction times for this circuit are displayed in Fig. 5 (see the graph on the right) and confirm that no biases or errors were attributable to these variations.

#### 3.1.3 Statistical controls and superquantum mechanics

While the preceding analyses were sufficient to demonstrate that the circuit’s qubits were entangled, the violation of Bell’s inequality with an *S* (CHSH) value exceeding the theoretical threshold of approximately 2.8284 (the square root of 8) raises the question of whether the observed entanglement and quantum correlations might reflect a superquantum model. This idea aligns with the superquantum framework proposed by Popescu and Rohrlich [64].

To rule out potential errors stemming from the *IBM Brisbane* real system that could affect the *S* value, two statistical controls were applied:

(1) *Comparison with a control circuit-2 (E2):* the nonlocal correlations, the *S* value, the concurrence, and the von Neumann entropy of the *experimental circuit* (E1) were compared to a *control circuit-2* (E2). The E2 circuit maintained all characteristics of E1, with one crucial modification: the random rotations were eliminated. This approach effectively blocked any noise distortions that could be attributed to these rotations.
(2) *Comparison with simulated and ideal conditions:* the results obtained with *IBM Brisbane* for E1 were compared with those from a *Qiskit* simulator and IBM under ideal conditions. This comparison enabled the identification and control of noise levels that could be attributed to the physical characteristics of the *IBM Brisbane* hardware.

If either of these noise sources could explain why the observed *S* value exceeded the theoretical threshold of 2.8284, the contrasts would allow us to adjust the *S* value to fall below this limit. However, if no such adjustments were required, the results would provide an initial indication that achieving values beyond the conventional quantum-theoretical threshold is possible, suggesting the existence of superquantum phenomena. Additional details are provided in Table 2.

**Table 2:** Quantum analysis of the degree of entanglement and the discrepancies between the real quantum system and the *Qiskit* ideal simulator with zero-error settings.

	*IBM Brisbane* (real system) Measurement error = 1.65 %		*Qiskit* (ideal simulator) Measurement error = 0 %	
Circuits	E1 (2QE = 0.304 %)	E2 (2QE = 0.304 %)	E1 (2QE = 0 %)	E2 E1 (2QE = 0 %)
*C*_*XX*_	0.9960	1.0000	0.9710	1.0000
*C*_*YY*_	−0.9960	−1.0000	−0.9710	−1.0000
*C*_*ZZ*_	1.0000	1.0000	1.0000	1.0000
*S* (CHSH)	2.9920	3.0000	2.9421	3.0000
Concurrence	0.8470	1.0000	0.8963	1.0000
von Neumann entropy	0.9980	1.0000	0.9996	1.0000
*S* difference	0.0499	0	0.0499	0

**Note:** 2QE= measurement error attributed to the CNOT gate.

The difference between Bell’s *S* (CHSH) value in the *IBM Brisbane* E1 circuit and the *S* value obtained from an ideal-condition simulation was 0.0499, indicating an approximate error rate of 5 %. However, this margin of error did not allow for adjusting the *S* value to the theoretical threshold of 2.8284. This outcome confirms that the physical system’s inherent errors cannot account for the observed *S* value exceeding this limit. Furthermore, when random perturbations were removed from the E1 circuit to create E2, the *S* values not only failed to decrease but instead increased to precisely 3. The differences between the circuits were nearly negligible. Thus, neither the system errors nor the errors attributable to rotational perturbations were sufficient to reduce the *S* value to 2.8284. This suggests that the system may be operating within a transitional zone of the superquantum domain—a framework that has thus far remained purely theoretical [63]. Far from being a limitation, this finding represents a significant advantage for our design. Paradoxically, it situates our system among the few real-world physical setups capable of producing violations of the Popescu-Rohrlich inequality, which sets the theoretical maximum threshold at the square root of 16 [64]. This intriguing possibility is explored in greater depth in the discussion section, where we propose new perspectives for integrating consciousness phenomena within the superquantum framework.

### 3.2 Empirical application of the *Q* coefficient

In this subsection, we applied a principal axis factor analysis to the keyboard matrices of participants using *tetrachoric* correlations. This method enabled the identification of vectors that revealed non-random structures or patterns capable of predicting a portion of the variance in correct and incorrect responses. Parallel analysis was employed to determine the number of latent factors to retain (i.e., hidden patterns or structures inferred from the data). For group E1-A, 40 factors were retained, explaining 23.54 % of the variance. For group E1-B, 39 factors were retained, accounting for 22.56 % of the variance in responses. For group C1-A, 38 factors were retained, explaining 22.82 % of the variance, and for group C1-B, 42 factors were retained, predicting 25.36 % of the variance.

To calculate the partial eta-squared coefficient, which is essential for estimating β, a series of 2 × 2 multifactorial ANOVA models were conducted. Additional details on this analysis are provided in subsection 3.4 of this report. For the current analysis, the partial eta-squared coefficient was 0.135. Using the aforementioned variances, the CHSH *S* values (1.000 and 2.992, respectively), and the concurrence values (0 and 0.8470), we computed the *Q* statistic (see (21), (22), (23), (24)).

(21) $$Q_{E1-A}=V_{k}\cdot \left(1+\beta \cdot C_{q}\cdot S\right)=0.2354\cdot \left(1+0.135\cdot 0.8470\cdot 2.992\right)\approx 0.3160$$

(22) $$Q_{E1-B}=0.2256\cdot \left(1+0.135\cdot 0.8470\cdot 2.992\right)\approx 0.3028$$

(23) $$Q_{C1-A}=0.2282\cdot \left(1+0\cdot 0\cdot 1\right)\approx 0.2282$$

(24) $$Q_{C1-B}=0.2536\cdot \left(1+0\cdot 0\cdot 1\right)\approx 0.2536$$

As demonstrated, applying entanglement (as shown in (21), (22)) results in a slight increase in the variance of keyboard responses. Specifically, for E1-A, the variance increased by approximately 31.60 %-23.54 %≈ 8.06 %, and for E1-B, by 30.28 %-22.56 %≈ 7.72 %. These increases in entanglement are proportional across both experimental groups, which consist of matched twin-pair participants. To determine whether the differences observed for E1-A were statistically significant, a Student’s *t*-test with 52 degrees of freedom per group was performed. The test yielded a *p-value* of 1 − *P*(*T* ≤ 14.038) < 0.01, leading us to conclude that the 8.06 % increase in E1-A could not be attributed to random fluctuations or associated sources of error. Similarly, for E1-B, the *p-value* was 1 − *P*(*T* ≤ 11.832) < 0.01, also indicating statistically significant results. This shows that the 7.72 % increase in E1-B likewise cannot be explained by random variability.

In conclusion, the *Q* statistic effectively incorporates the effects of entanglement, which have a measurable and significant cognitive impact. These findings suggest that entanglement plays a key role in quantum-like learning processes.

### 3.3 3D correlations for validating the *Q* coefficient

**Fig. 6:**
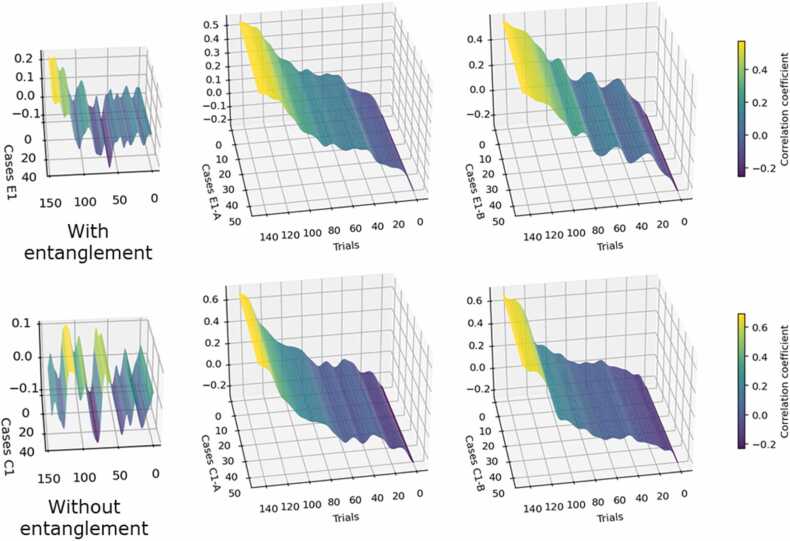
**3D Multilinear correlations between keyboard response matrices and collapse matrices (labeled as A and B).** The correlation trends should be examined to assess whether any form of learning has occurred. Specifically, an increase in correlations toward the final columns (out of 144) would indicate such learning. The left section of this figure analyzes the correlations between the keyboard response matrices for twin pairs exposed to E1-A and E1-B. In the “with entanglement” graph, the correlations exhibit less randomness compared to the “without entanglement” graph, providing initial evidence that may support potential effects attributed to entanglement.

The procedure for validating or verifying the *Q* coefficient is detailed in Appendix B of this report. Fig. 6 presents topographic maps illustrating the multilinear correlations between keyboard responses and the collapse matrices. These maps allowed us to assess the validity of the *Q* coefficient results by examining whether the correlations increased progressively throughout the experimental trials.

The results confirmed that collapses A and B of E1 exhibited visibly higher levels of synchronicity compared to collapses A and B derived from C1. This finding supports the possibility that entanglement effects were correlated with participants’ responses. The increasing trend in the magnitude of correlations between the matrices shown in Fig. 6 indicates that participants demonstrated a form of nonlocal learning, which would not be expected through purely rational processes. Importantly, the two smaller graphs on the left side of Fig. 6 illustrate the correlations between the keyboard responses of E1-A and E1-B, as well as C1-A and C1-B (i.e., the correlations between the responses of twin-pair participants and their siblings). Focusing on these two graphs, the correlations for E1 display a clear upward trend, suggesting the presence of a synchronous connection or entanglement between the twins. If this effect were attributable to other sources of variation unrelated to entanglement, a similar pattern would be observed in the C1 correlations. However, this is not the case. The “without entanglement” graph shows a more random and unstable correlation pattern compared to the top graph for E1. These observations provide further evidence that entanglement may have influenced participants’ cognitive responses, consistent with the 2 % *Q* estimation. While the effect size is small, the difference is statistically significant.

### 3.4 Multiple regression models

#### 3.4.1 Descriptive statistics

Table 3, Table 4 summarize the descriptive statistics of the variables in this study. Table 4 shows the descriptive statistics and the multifactorial analysis of variance of the hit/miss matrix with the total scores. All conditions of statistical normality and homogeneity of variances were met.

**Table 3:** Descriptive analysis of biological markers and brain *regions of interest* (ROIs) assessed through electroencephalography.

	Group 0 without entanglement (paired A-B twins)		Group 1 with entanglement (paired A-B twins)	
	Means (SD) A	Means (SD) B	Means (SD) A	Means (SD) B
BDNF	19.57 (5.275)	20.72 (5.865)	20.42 (3.177)	20.34 (2.714)
FFA	0.320 (0.065)	0.308 (0.068)	0.305 (0.024)	0.304 (0.022)
Alpha-Amylase	54.30 (6.818)	54.81 (7.142)	51.42 (2.349)	51.98 (2.179)
Frontal ROI	−1.48 (20.339)	2.27 (21.480)	−0.48 (24.378)	0.694 (24.473)
Parietal ROI	−0.67 (8.958)	0.90 (8.512)	−0.29 (7.686)	−0.17 (7.762)
Coronal ROI	1.16 (10.253)	−0.72 (10.490)	−0.13 (9.128)	0.35 (9.391)
Temporal ROI	−1.39 (10.292)	1.33 (12.398)	0.27 (14.003)	0.20 (13.701)
Occipital ROI	0.12 (9.509)	0.42 (10.382)	−0.32 (9.871)	0.104 (9.811)

**Note:** SD= Standard deviation; BDNF= Brain-Derived Neurotrophic Factor; FFA= Free Fatty Acids; ROI= region of interest.

**Table 4:** Descriptive analysis of variance using hit/error matrices for paired twins.

Factors	Fisher’s *F* (*df*)	*P-values*	Partial η^2^	Means (Standard deviations)
Paired effects	2.088 (1)	0.151	0.020	A_0_= 80.83 (5.480) A_1_= 84.85 (6.004) B_0_= 81.869 (5.027) B_1_= 84.358 (5.485)
Interaction effects	16.290 (1)	< 0.001	0.135	
Entanglement effects	9.546 (1)	0.003	0.084	

The results in Table 4 show that the entanglement predicted 8.4 % of the variance. However, if there are significant interaction effects, this explained variance is meaningless because we have to replace it with the variance of the interaction. In the case of the interaction with the matched twins (pairwise effects), this percentage prediction increases to 13.5 %. This is consistent with what we used in the *Q* coefficient.

#### 3.4.2 Regressions applied to the EEG results

**Fig. 7:**
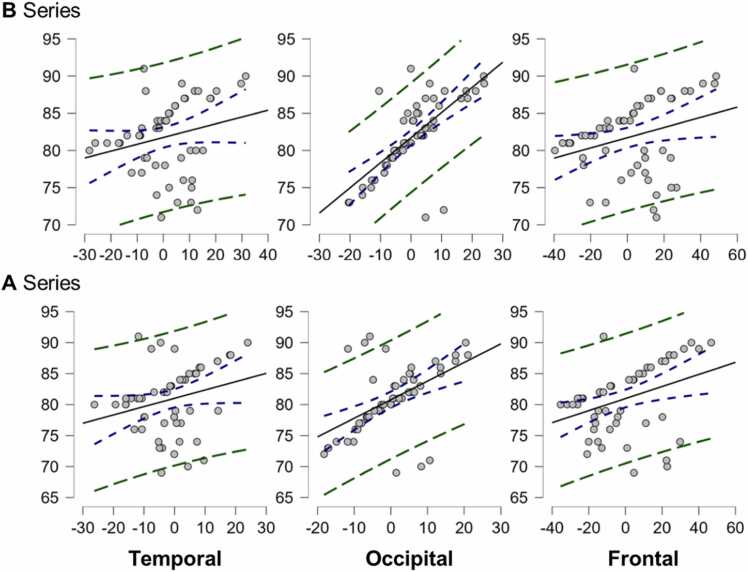
**Regression lines corresponding to the EEG results (group 0).** Regression lines for the temporal, occipital, and frontal lobes of the twins assigned to group 0, which exhibited no entanglement. These lobes were retained as predictor variables using the backward stepwise method, a technique well-suited for developing multiple regression models as it prioritizes parsimony and adopts a conservative approach favoring the null hypothesis.

**Fig. 8:**
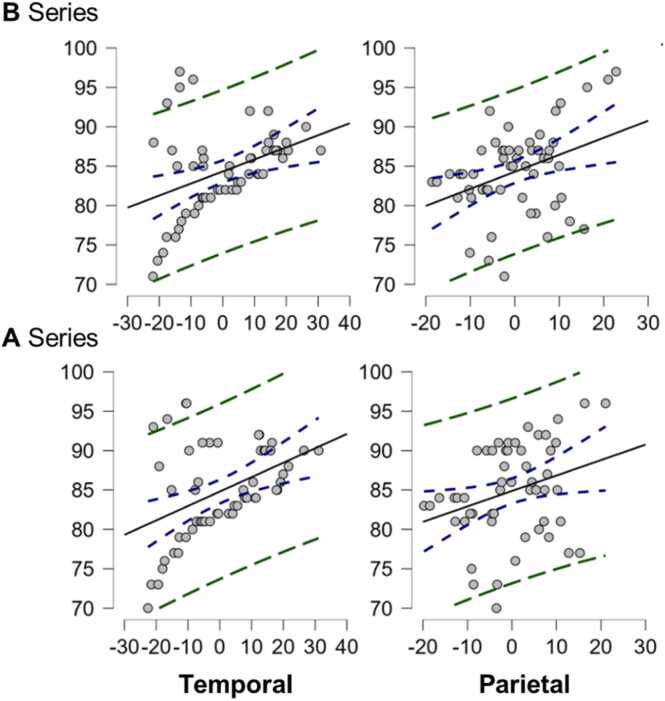
**Regression lines corresponding to the EEG results (group 1).** Regression lines for the temporal and parietal lobes in group 1 with entanglement. Predictor variables were determined using the backward stepwise regression method, which is methodologically recognized as the most conservative approach for developing a parsimonious model.

The EEG results were analyzed qualitatively and quantitatively. The quantitative analyses are the linear predictions of each ROI with respect to the performance level of the twins. In Fig. 7, Fig. 8, we present the regression lines with predictions relating the electrochemical activity of each region to quantum-like learning only for the regions that contributed significant changes.

The results of group 0 (Fig. 7) for a multiple regression model using the ordinary least squares parameter estimation criterion and the backward stepwise method on the five initially included ROI variables defined a model that retained only the predictors corresponding to the electrochemical activity of the temporal, occipital, and frontal areas. The criterion variable was the participants' total number of hits in quantum-like learning. For the A-twins the following results were obtained: intercept= 80.724, β_1_= -0.166 (error= 0.097, standardized= −0.310), β_2_= 0.311 (error= 0.077, standardized= 0.540) and β_3_= 0.109 (error= 0.045, standardized= 0.405). In this case, the multiple correlation coefficient was 0.591, with an adjusted *R*^2^ of 31 % (*p-value*<0.01). The *Root Mean Square Error* (RMSE) for this model was 4.553, which gives a relatively small percentage error when we standardize, approximately 3.16 %. For B-matched twins, we obtained an intercept= 81.724, β_1_= -0.101 (error= 0.066, standardized= −0.250), β_2_= 0.345 (error= 0.052, standardized= 0.714), and β_3_= 0.059 (error= 0.037, standardized= 0.250). The multiple correlation coefficient was 0.718, with an adjusted *R*^2^ of 48.5 % (*p-value*<0.01) and RMSE= 3.606, implying an error of 2.50 %, which is very satisfactory.

**Fig. 9:**
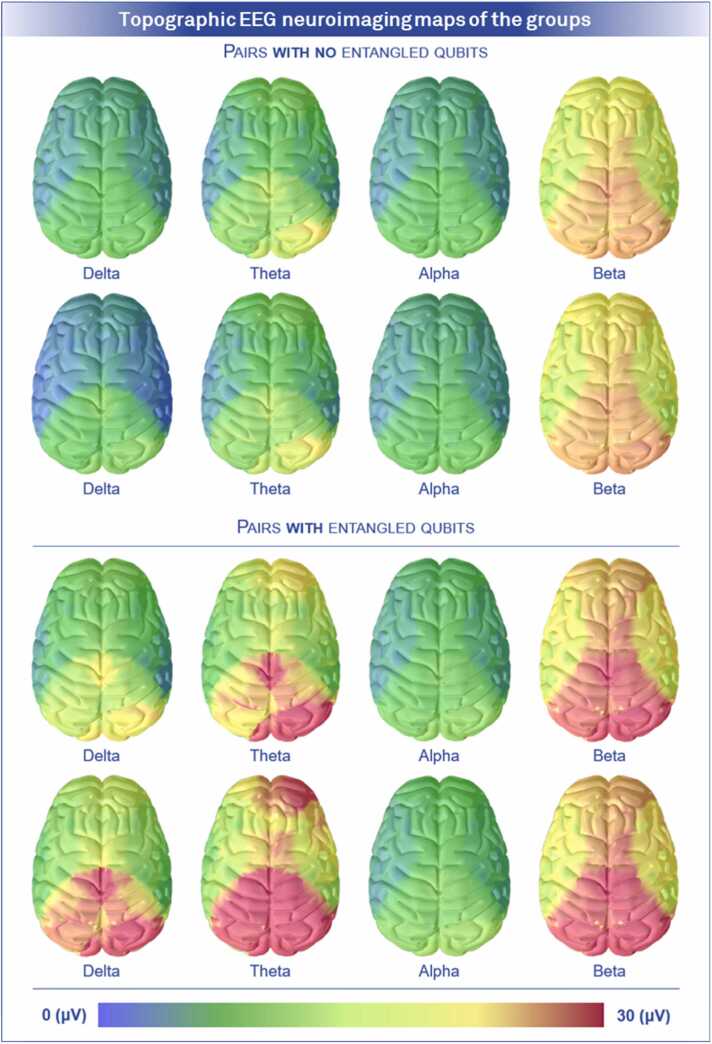
**Topographic EEG neuroimaging maps of the groups.** Topographic maps of the twins' electrochemical activity during responses to each of the 144 trials. Measurements are reported in microvolts, differentiating between conditions with and without quantum entanglement. In each block, the first row represents twin A, while the second row corresponds to the paired twin B.

The results for group 1 (Fig. 8) were obtained using the same criteria as in the previous paragraph. For the A-twins, the results were as follows: intercept= 84.826, β_1_= 0.227 (error= 0.050, standardized= 0.529) and β_2_= 0.280 (error= 0.077, standardized= 0.425). The multiple correlation coefficient was 0.594, with an adjusted *R*^2^ of 32.87 % (*p-value*<0.01) and RMSE= 4.927 (error= 3.42 %). For B twins, the results were: intercept= 84.211, β_1_= 0.211 (error= 0.046, standardized= 0.527) and β_2_= 0.302 (error= 0.067, standardized= 0.517). The multiple correlation coefficient was 0.627, with an adjusted *R*^2^ of 36.80 % (*p-value*<0.01) and RMSE= 4.359 (error= 3.03 %). Qualitatively, we show the neuroimages in Fig. 9, which are microvolt topographic maps of the activations at the time of response to each of the trials in each of the brain regions evaluated.

In Fig. 9, electrochemical activations were predominantly observed in the occipital, parietal, and frontal regions, with the posterior occipital area displaying the highest magnitudes. This pattern was consistent across both groups; however, in group 1, mental states at the precise moment of decision-making showed significantly higher activation compared to those in the other group. Fig. 9 also highlights that theta mental states exhibited activations exclusively in group 1, which notably was the group exposed to the entanglement condition. Since theta waves are typically associated with mental states related to sleep, this suggests a polarization in the activations among the twins who participated in experiments involving entangled qubits. Although the reasons behind these anomalies are not yet clear, potential explanations for this phenomenon will be addressed in the discussion section. In summary, the temporal, occipital, and frontal lobes in group 0 were key predictors of the twins’ total correct responses. For group 1, however, significant correlations were limited to the temporal and parietal lobes.

#### 3.4.3 Regressions applied to the biomarkers

**Fig. 10:**
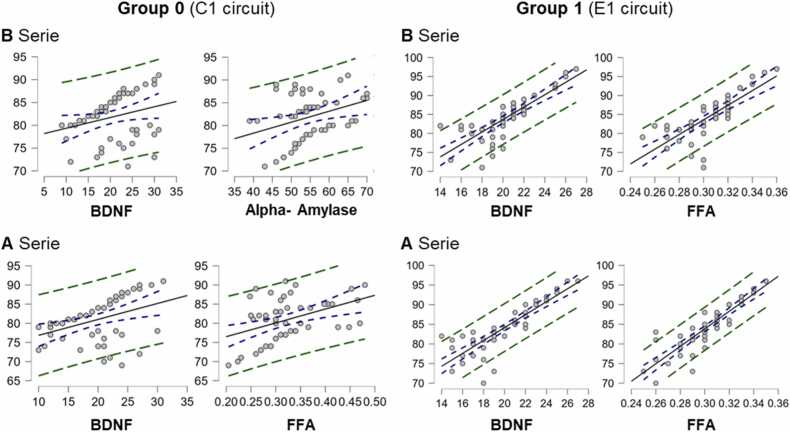
**Regression lines corresponding to the biomarkers.** Regression models predicting performance levels in quantum-like learning based on biomarkers related to conscious experience measured in this study. The regression lines are presented for group 0 (no quantum entanglement) and group 1 (with the entanglement condition).

In this subsection, we present the extent to which the three biomarkers used in this study were able to predict performance levels on quantum-like learning tests. We used the same system and criteria as in the other multiple regressions. Fig. 10 shows the regression lines for these analyses.

In group 0 and for A-twins, the results retained BDNF and FFA biomarkers: intercept= 61.561, β_1_= 0.406 (error= 0.120, standardized= 0.391) and β_2_= 35.347 (error= 9.633, standardized= 0.422). The multiple correlation coefficient was 0.581 and the adjusted *R*^2^ showed an explained variance of 31.2 % (*p-value*<0.01), with RMSE= 4.546 (error= 3.16 %). Focusing on the biomarker of most interest to us, related to plasticity, we saw that BDNF contributed an explained variance of 15.29 %. On the other hand, for the B twins, keeping BDNF and alpha-amylase, the results were: intercept= 64.818, β_1_= 0.217 (error= 0.110, standardized= 0.253) and β_2_= 0.229 (error= 0.090, standardized= 0.325). The multiple correlation coefficient was 0.425 and the adjusted *R*^2^ showed an explained variance of 14.80 % (*p-value*= 0.013 <0.05), with RMSE= 4.641 (error= 3.22 %). The explained variance for BDNF alone was not significant in this case.

In group 1 and for A-twins we obtained a model including only BDNF and FFA with the following results: intercept= 26.448, β_1_= 0.805 (error= 0.163, standardized= 0.426) and β_2_= 137.781 (error= 21.429, standardized= 0.554). The multiple correlation coefficient was 0.931 and the adjusted *R*^2^ showed an explained variance of 81.6 % (*p-value*<0.01), with RMSE= 2.239 (error= 1.55 %). BDNF contributed 18.15 % of the explained variance. For B-twins the results were: intercept= 33.805, β_1_= 1.034 (error= 0.233, standardized= 0.512) and β_2_= 96.724 (error= 28.494, standardized= 0.392). The multiple correlation coefficient was 0.850 and the adjusted *R*^2^ showed an explained variance of 71.7 % (*p-value*<0.01), with RMSE= 2.950 (error= 2.05 %). The explained variance for BDNF was 26.2 %.

These findings suggest that individuals with a higher predisposition for plasticity (as indicated by BDNF levels) tend to achieve better performance levels compared to those with lower plasticity. This relationship was quantified at 26.2 % in the group of twins exposed to entanglement, leading us to hypothesize that the configuration of stimuli involving entangled qubits may have incorporated a quantum structure that influenced cognitive information processing.

Additionally, FFA levels were found to predict performance outcomes, reinforcing the hypothesis that a greater energy consumption capacity may facilitate learning processes. This interpretation aligns with the hypothesis proposed by Marwaha and May [52], who linked entropy to the availability of environmental energy as a predictor of anomalous cognitive phenomena.

## 4 Discussion

The findings of our study support the initially proposed hypotheses (1), (2), (3), and (4). Collectively, these hypotheses imply acceptance of the idea that qubit entanglement applied to the configuration of stimulus contingencies in our experiments influences unconscious cognitive decisions recorded under conditions of implicit learning or quantum-like learning.

### 4.1 Acceptance of hypotheses and evidence analysis

For hypothesis (1), twins with a higher predisposition to plasticity demonstrated better performance levels, with explained variance ranging from 15.29 % (without entanglement) to 26.2 % (with entanglement). This suggests that entanglement enhances the effects attributable to plasticity. This finding aligns with the NPT, which posits that plasticity levels play a critical role in the production of anomalous cognitions. It also supports the hypotheses of Han and Reber [36] and Han et al. [37], which emphasize that brain plasticity operates globally rather than being confined to specific regions.

In the case of hypothesis (2), statistical evidence also supports its validity. Mental activation states across most brain regions—except for the coronal region of the parietal lobe—positively correlate with correct responses in quantum-like learning tasks. This indicates that anomalous cognitions are more likely to occur in states of activation. For the entanglement group, significant activations were observed in theta states, a type of rest-sleep state. The combination of both mental states within the entanglement group raises questions about whether the polarization of molecular functioning in the brain’s electrochemical activity could serve as a biological marker of entanglement effects beyond cognitive domains. There is currently no published scientific literature in indexed journals addressing this phenomenon. Thus, we cannot determine whether this observation is isolated or replicable in future research. While we must approach this finding cautiously, it opens the door to speculation and further inquiry. Notably, this bipolarity in mental states is consistent with the logic of entanglement, where the coherence of entangled particles produces alternating systematic changes. For instance, when one electron is in state 1, its entangled counterpart might be in state 0, provided the entanglement has been prepared accordingly. Consequently, linking this physiological bipolarity observed in EEG results to entanglement is plausible. This notion of connecting global plasticity with implicit learning processes aligns with the review by Gonçalves et al. [33] on the global functioning of conscious experience. Combining our results with these insights leads us to propose that, if Wahbeh et al. [79] work on fundamental consciousness is accurate, plasticity may play a significant role and could serve as a biological marker for progress in this area.

Hypothesis (3) centers on the *Guppy Effect*, which posits that associating two stimuli contingently increases the likelihood of correct responses in a cognitive learning process. Aerts and Sozzo [3] proposed that conceptual entanglement in such associations could enhance the attribution of concepts to prototype pairs. While their idea was initially grounded in the cognitive foundations of linguistics, our study tested the *Guppy Effect* by analyzing the impact of quantum entanglement on qubits. If entanglement influenced the stimulus configuration, it would lead to improved performance among twins exposed to entanglement during the experiments—our findings confirm this possibility. The combined effects of entanglement and genetic pairing among twins explained 13.5 % of the total variance in correct responses, with entanglement accounting for 8.4 % of this variance. The primary implications and significance of these findings are twofold: (a) our data strongly supports Aerts and Sozzo’s [3] hypothesis that conceptual entanglement underpins the *Guppy Effect*, and (b) quantum entanglement significantly enhances learning efficiency when applied to the configuration of stimulus contingencies. These contingencies serve as the foundation for drawing such inferences. Moreover, these results suggest exciting new opportunities to investigate the experimental impact of quantum entanglement in contexts similar to ours, particularly in linguistic phenomena involving implicit learning processes.

Notably, our findings also align with *Google’s* official announcement of a technology that connects human brains to quantum computers under entangled conditions [47]. However, unlike *Google*, we achieved these results independently—without utilizing their technologies or patents. This invites critical reflection on the revolutionary potential of our discoveries and their implications for neuroscience, learning, and consciousness studies.

Hypothesis (4) is the most innovative, as it suggests developing a statistical coefficient that integrates variations based on both classical and quantum probabilities. Our *Q* coefficient demonstrates that this hypothesis is feasible, practical, and potentially valuable for research in theoretical physics and molecular biology using quantum mechanics to investigate consciousness. The simplicity of the *Q* coefficient does not diminish its value; methods that are easy to apply tend to be easier to reproduce, making *Q* a robust statistical tool. However, researchers intending to use the *Q* coefficient should consider several important factors:

(1) *Factorization considerations.* Correlation matrix structures were analyzed using factor analysis, which requires large samples to yield statistically stable eigenvalues. Our sample size of 53 cases fell short of these recommendations. While this does not invalidate the *Q* coefficient, researchers should note that larger samples increase the likelihood of detecting non-random response patterns. Additionally, we used the principal axes method, which is suitable for identifying covariation patterns. However, researchers focusing on latent variables should consider robust maximum likelihood methods to maximize the reproducibility of correlation or covariance matrices. These methodological notes are intended to guide future contributions or analyses involving the *Q* coefficient.
(2) *Quantum concurrence and CHSH (S) considerations.* The *Q* coefficient is only meaningful in studies designed to approximate quantum entanglement in cognitive or macroscopic phenomena. If the necessary methodological conditions are absent, using *Q* is inappropriate, as the results will remain invariant relative to classical explained variance. While this may seem obvious, many studies attempting to validate anomalous cognition phenomena, such as precognition, were flawed due to methodological limitations that undermined their internal validity [57]. As with any statistical coefficient, the properties of *Q* depend on the quality of the measurements, which requires rigorous experimental methodologies. Interpretively, while *Q* incorporates concurrence and *S*, this does not imply that conscious experience originates from quantum mechanics. It also does not suggest that consciousness inherently operates quantum-mechanically. Instead, quantum measurements indicate contextual and quantum-like elements (e.g., stimulus contingencies) influencing the twins’ conscious states.
(3) *Beta coefficient estimation considerations.* In the *Q* framework, β is a parameter that modulates quantum effects on variance. It is essential to derive β from participants’ accuracy/error matrices, not keyboard response matrices. Using the latter inflates β, leading to Type I errors. Instead, β should be calculated directly from accuracy/error matrices. While a factorial analysis could be performed on this matrix, robust methods such as partial eta squared should be prioritized when available, as they correct for issues associated with matched pair designs. In our case, β was estimated at 13.5 %, which reflects the explained variance attributable to experimental conditions, including entanglement and matched pair controls.

Future researchers should take these methodological considerations into account when applying *Q* in experimental designs to explore its predictive capabilities and broader applicability.

### 4.2 Superquantum vs. quantum effects

Since our Bell’s *S* exceeded the theoretical maximum threshold applicable to real physical systems, readers may question whether the results from the E1 density matrix analysis fall within the domain of superquantum phenomena [64]. Our position on this is clear: while we cannot claim that the conditions and properties of qubit entanglement are superquantum, we can make the following points. First, we examined whether the *S* value obtained in our circuit (applied to a real physical system, *IBM Brisbane*) might be due to rotations or logical gates introducing noise into E1. The results showed this was not the case. In noise-free conditions, the *S* indicator increased to 3, a value firmly within the superquantum domain. Second, we verified that this outcome could not be attributed to characteristics of the real physical system *IBM Brisbane* itself. To do this, we replicated the calculations and circuits using an ideal simulator with errors set to zero. When running E1 in this perfect simulator, the results closely matched those from our experiments. However, the differences between the ideal and real conditions (in the experimental *S* values) did not allow us to predict the observed increase in *S* beyond the theoretical threshold.

Based on these validations, we have reason to suggest that *IBM Brisbane*, in ways we do not yet understand, produced variations in *S* slightly exceeding the usual quantum limits. If this entanglement is indeed superquantum rather than quantum, this would have two major implications for interpreting our findings:

(1) We would need to assume that *IBM Brisbane*, via our circuit, induced states in the qubits that emergently caused this variation through unknown perturbations that we could not identify. This aligns with Gisin et al.’s [32] findings, which demonstrated that the quantum CHSH limit can be surpassed. Such emergent behavior would place Popescu’s [63] superquantum framework into the realm of applied experimentation in consciousness research. While innovative, this aligns with the growing number of methods and criteria for applying quantum mechanics to non-quantum processes [71].
(2) Accepting (1) would mean acknowledging that nonlocality precedes uncertainty, rather than the other way around. This idea, proposed by Popescu [63], is compatible with the fundamental consciousness hypothesis [79]. If so, our evidence would align with this research direction. However, within the scope of our study, we do not yet have enough evidence to demonstrate that our experimental results go beyond quantum mechanics.

### 4.3 On quantum limitations

The limitations of this study, some of which have already been addressed, focus on the broader question of whether quantum-level phenomena can be generalized to non-quantum or cognitive levels. The integration of quantum mathematics into real-world processes has sparked considerable debate for several reasons, notably:

(1) There is a mathematical issue of decoherence when applying quantum principles to non-quantum phenomena [77].
(2) Multiple interpretive frameworks exist—most of which are based on scientific consensus—but it is unclear which should apply when combining quantum and non-quantum methodologies [38].
(3) The philosophical “hard problem” remains: Why should quantum probabilities apply to systems in reality that are not inherently quantum [59]?

In addressing these critiques, we note that the *Q* coefficient specifically seeks to address point (1); the other issues must be discussed scientifically once there is sufficient evidence to evaluate the success or failure of using quantum models in non-quantum contexts. We argue that these questions should not be addressed or judged at this stage of the research and are beyond the scope of this report. Attempting to do so would be as premature as expecting the inventors of the steam engine to justify its intellectual and technological revolution before its societal impact was understood. Such questions are logically unanswerable: we understand the meaning of events only after they occur, not before. Thus, we see no objective value in prematurely judging the *Q* coefficient’s validity. Its adoption by other researchers should be encouraged, and its functionality should be assessed statistically. Only then will we be in a position to offer a deeper analytical and philosophical judgment.

Regarding limitation (1), we emphasize the methodological framework underpinning the inference of entanglement effects. It is not only that the E1 qubits exhibited entangled states; somehow, these states quantumly influenced the collapse matrix. Since this matrix was used to configure the stimuli in our experiments, we have reason to assert that a quantum phenomenon at this stage altered participants’ performance levels. The decision to use twins was made before the study began, based on the well-documented phenomenon of electrochemical synchronicities observed in identical twins [76]. If such a phenomenon had any effect in our study, as shown in Table 4, it did not yield statistically significant results (*p-value*= 0.151) and thus did not directly impact our analyses. While we have not mathematically resolved the problem of decoherence, we have an empirically testable procedure that overcomes it—even if we do not fully understand how. Further research is needed to explore the revolutionary potential of these findings.

### 4.4 Conclusions

Our findings indicate that certain learning effects violate locality conditions and exhibit predictable cognitive behavior influenced by quantum entanglement in monozygotic twins, explaining 13.5 % of the variance in performance levels. This explained variance may also reflect a subtype of *Guppy Effect* [62], potentially representing one of the first pieces of evidence that cognitive entanglement plays a role in this type of learning through the contingencies discussed earlier. Furthermore, this variance cannot be attributed to other known effects that might have distorted our results. **Hence, we can conclude that entanglement significantly enhances the efficiency of unconscious learning processes, potentially boosting the accuracy and effectiveness of cognitive performance.**

Using our newly developed *Quantum-Multilinear Integrated Coefficient* (*Q*), the non-random structures in participants’ responses increased by approximately 31 %, with about 8 % of the variance predictably explained by entanglement. This suggests that quantum entanglement in cognitive processes, especially in the realm of anomalous cognition, introduces variations that can predict responses. Our findings support research linking conscious experience with quantum mechanics. We propose the *Q* coefficient for use in future studies and encourage the international research community to adopt or refine it to advance this line of inquiry.

Another important biological conclusion concerns neural plasticity. The fact that BDNF results account for approximately 26.5 % of the variance in cognitive performance supports the theoretical basis of the *Nonlocal Plasticity Theory* (NPT) proposed by Escolà-Gascón [24]. Our findings indicate that high plasticity levels are associated with nonlocal cognitive mechanisms involved in implicit learning. Future research on precognition should consider brain plasticity as a potential biomarker. While it may not resolve the hard problem of consciousness [15], it provides a pathway for empirically testing this phenomenon and developing applications to enhance survival. This also corroborates previous evidence of anomalous cognitions operating beyond the ontological boundaries traditionally set by orthodox science. Such findings challenge conventional scientific knowledge, not through mystical interpretations but through phenomena that transcend current epistemological frameworks.

## Funding statement

This research was conducted with funding awarded by the *Society for Psychical Research* (10.13039/100009798SPR), United Kingdom, as part of its 2024 competitive project grant program. The principal investigator and author of this report wishes to express profound gratitude to the SPR for their trust and support in advancing this research endeavor. Similarly, this work was also supported by the *Catholic Church* through its healthcare infrastructure and the *Pontifical University* to which the author is affiliated, an institution established by the *Holy See*, *Vatican City State*.

## Author statement

This research was conducted entirely by *Prof. Dr. Álex Escolà-Gascón*, who is the sole author of this work.

## Ethical statement

The *Committee for Ethical Guarantees of the Present University* conducted a favorable review of the research protocol. Each participant provided informed consent, which elucidated the study’s objectives and the assessment tests employed. Participation was strictly voluntary, with participants retaining the right to withdraw from the study at their discretion. Additionally, all collected data were processed with utmost confidentiality and anonymity.

## CRediT authorship contribution statement

**Álex Escolà-Gascón:** Writing – review & editing, Writing – original draft, Visualization, Validation, Software, Resources, Project administration, Methodology, Investigation, Funding acquisition, Formal analysis, Data curation, Conceptualization.

## Declaration of competing interest

There are no known conflicts of interest associated with this publication.

## Data Availability

The data will be available upon request to the author of the manuscript, provided that properly justified reasons are given. The author reserves the right to decline any data requests that may involve a conflict of interest.

## Appendix A Definition of fundamental concepts in quantum computing

(1) **What is a quantum computational circuit?**

A quantum computational circuit is a model used to perform calculations by leveraging the principles of quantum mechanics. It consists of qubits (quantum units of information) and quantum gates (operations that manipulate qubits). While similar in concept to classical logic circuits, quantum circuits take advantage of unique quantum properties like superposition and entanglement, allowing them to process information more efficiently for certain types of problems.

(2) **What is a qubit?**

A qubit (*quantum bit*) is the fundamental unit of information in quantum computing, similar to a classical bit. However, unlike a classical bit, which can only be 0 or 1, a qubit can exist in both states simultaneously due to a property called superposition. This allows quantum computers to perform multiple calculations at once, making them potentially far more powerful than classical computers for specific tasks.

(3) **Why do qubits behave differently from classical bits?**

Qubits follow the rules of quantum mechanics, which give them properties that classical bits don’t have: (a) *superposition*—a qubit can exist in multiple states at the same time, rather than being strictly 0 or 1; (b) *entanglement*—two or more qubits can become linked, meaning that a change in one instantaneously affects the other, no matter how far apart they are; and (c) *quantum interference*—qubits can combine and cancel out different probabilities, helping improve the efficiency of certain quantum algorithms.

(4) **What are quantum gates and how do they work?**

Quantum gates are operations that change the state of qubits in a quantum circuit. They are similar to classical logic gates (like *AND* and *OR*), but instead of switching bits between 0 and 1, they manipulate qubits in superposition. Quantum gates are also reversible, meaning no information is lost in the process—an important distinction from classical computing.

(5) **What is a*****Hadamard*****gate and what does it do?**

The *Hadamard* gate (*H*) is one of the most fundamental quantum gates because it creates superposition. If a qubit starts in state 0 or 1, applying a *Hadamard* gate transforms it into a state where it is equally likely to be either. This is essential in quantum computing, as it allows a single computation to explore multiple possibilities at once.

(6) **What is a CNOT gate and why is it important?**

The CNOT (*Controlled-NOT*) gate is a two-qubit quantum gate that applies a conditional operation: if the first qubit (control qubit) is 1, it flips the second qubit (target qubit). If the control qubit is 0, the target qubit remains unchanged. This gate is crucial for quantum entanglement, a key feature used in quantum algorithms and quantum cryptography.

(7) **What are*****Pauli*****operators, and how do they relate to quantum noise?**

*Pauli* operators describe how a qubit’s state can rotate in different directions. In the presence of quantum noise, these operators help model how external factors can disrupt qubit information. This understanding is critical for quantum error correction, which aims to make quantum computers more reliable.

(8) **What happens when a qubit “collapses” to 0 or 1?**

Before measurement, a qubit exists in a superposition of 0 and 1, meaning it holds a probability of being in either state. However, when a qubit is measured, it “collapses” into a definite state—either 0 or 1—and loses its superposition. This collapse is a fundamental part of quantum mechanics.

(9) **How do we mathematically describe a quantum circuit?**

Quantum circuits are typically represented using *bra-ket* notation, which describes qubit states as vectors in an abstract mathematical space. Additionally, quantum gates are expressed using unitary matrices, and quantum circuit diagrams are used to visually map out the sequence of operations.

(10) **What is the difference between a real quantum computer and a quantum simulator?**

A *real* quantum computer uses physical qubits made from quantum particles (such as electrons or photons) to perform calculations. Because it operates on true quantum principles, such as superposition and entanglement, it can solve certain problems exponentially faster than classical computers. In contrast, a quantum *simulator* is a software program that runs on a classical computer to imitate the behavior of a quantum system. While it can simulate qubits and quantum gates, it is limited by the constraints of classical computing and cannot achieve the same speed or efficiency as a real quantum computer.

## Appendix B Verification of the *Q* coefficient

Since the *Q* coefficient is a newly proposed statistic designed to integrate quantum functionality with general mechanics, we also tested in this study whether the structures of participants’ keyboard responses align with the structures of the A and B collapse matrices. To empirically confirm that entanglement influences participant responses and validate the *Q* coefficient, it is crucial to establish a covariant structure between the keyboard responses and the collapse matrices. This step is essential to empirically substantiate the interpretability of the *Q* coefficient; without it, its interpretation would lack rigor and become arbitrary.

This analysis can be approached through various methods. One viable approach involves calculating multilinear correlations between the participants' keyboard response matrices and the A and B collapse matrices generated by E1. If the analysis succeeds, we should observe topographically visualized correlations greater than 0 that progressively increase in magnitude. However, correlations greater than 0 alone are insufficient to confirm entanglement effects. As noted earlier, the topographic map must display progressively increasing correlations > 0 to substantiate the presence of entanglement. This requirement is grounded in a fundamental logic: if implicit learning is functional, and the eigenvalue in the success/error matrices is high, participants’ successes should concentrate toward the later stages of the 144-trial sequences, rather than the earlier ones. While Escolà-Gascón [24] observed this clustering of successes toward the end of the sequence, the critical point here is that our comparative analysis should reveal significant differences in the distribution of successes between the C1 circuit group and the E1 circuit group with entanglement. If our hypothesis is correct, the increasing magnitude of correlations between matrix vectors should manifest visually, consistently remaining greater than 0.

To accurately capture this progressive increase, computational visualization must be presented in 3D. Without this dimensionality, the incremental growth in correlation magnitudes would not be discernible. By adding a *z*-axis to the map, we can represent the progressive increase in correlations over time. The details of this *z*-axis will be elaborated in the results section, providing readers with a clear conceptual link between the theoretical framework discussed in this subsection and the execution of the full procedures applied to real data.
